# Endoscopic transorbital approach for left anterior clinoidectomy, optic canal decompression and spheno-orbital meningioma resection

**DOI:** 10.3171/2025.1.FOCVID24199

**Published:** 2025-04-01

**Authors:** Tristan Brunette-Clement, Evan Kalin-Hajdu, Pascal Lavergne

**Affiliations:** 1 Department of Surgery, University of Montréal, Québec;; 2 Division of Neurosurgery, Montreal Sacred Heart Hospital, Montréal, Québec;; Divisions of 3 Ophthalmology, Maisonneuve-Rosemont Hospital, Montréal, Québec, Canada and; 4 Neurosurgery, Maisonneuve-Rosemont Hospital, Montréal, Québec, Canada

**Keywords:** endoscopic transorbital approach, spheno-orbital meningioma, optic canal decompression, anterior clinoidectomy

## Abstract

Endoscopic transorbital approaches have been increasingly used to treat lesions of the middle fossa, such as sphenoid wing meningiomas or cavernous sinus disease, eschewing the cosmetic and functional consequences of a frontotemporal craniotomy, as well as technical shortcomings of endoscopic endonasal approaches. In this video, the authors present a case of a spheno-orbital meningioma for which an endoscopic transorbital approach was used for tumor resection, optic canal decompression, and anterior clinoidectomy. The advantages of this approach are demonstrated, particularly the direct, shorter distance to target along the axis of the middle fossa, which avoids neurovascular structures, brain retraction, and temporal muscle manipulation.

The video can be found here: https://stream.cadmore.media/r10.3171/2025.1.FOCVID24199

## Transcript

This video will demonstrate the use of the endoscopic transorbital approach for left anterior clinoidectomy, optic canal decompression, and spheno-orbital meningioma resection.

### 0:30 Introduction.

In recent years, there has been a shift toward minimally invasive neurosurgery for certain skull base pathologies, focusing on more discreet incisions, smaller craniotomies, and minimal brain retraction. Endoscopic transorbital approaches have been increasingly used to treat lesions of the anterior and middle fossae.^1,2^ Lesions such as sphenoid wing meningiomas, trigeminal schwannomas, or cavernous sinus disease, which would traditionally require a frontotemporal craniotomy, can be treated with this minimally invasive approach.^3–7^ This approach avoids the cosmetic and functional consequences of temporal muscle atrophy, frontalis palsy, and masticating pain, which can occur with a frontotemporal craniotomy, as well as the potential parenchymal and venous injury.^6^ The endonasal approach has been used as an alternative to reduce these complications and has become a workhorse of the skull base surgeon to access the anterior fossa. However, it remains challenging to reach middle fossa pathology because of the long distance to targets, difficult angles, and intervening vascular structures which must be crossed.^8^ Endoscopic transorbital surgery was developed as an alternative to overcome these shortcomings of traditional approaches.^1,4^ Various corridors have been described.^9^

### 1:41 Surgical Technique.

Our technique involves incising the superior eyelid, transecting the orbicularis muscle laterally to the orbital rim, taking great care not to injure the levator muscle, deep to the orbital septum. Endoscopy-assisted subperiorbital dissection exposes the lateral orbital wall, all the way from superior to inferior orbital fissures. Drilling of the orbital wall and roof allows access to the middle and anterior fossae. Depending on the pathology, the optic canal, anterior clinoid, or lateral wall of the cavernous sinus can be reached. No orbital osteotomy is necessary in most cases.

### 2:14 Clinical Presentation.

The patient was in her late 50s. She presented with exophthalmos and loss of vision in her left eye, which were confirmed on examination. Only light and movement could be perceived in her left eye.

### 2:22 Preoperative Imaging.

Preoperative imaging revealed a hyperostotic lesion of the sphenoid wing with extension to the optic canal and anterior clinoid, but minimal intracranial extension, typical of a spheno-orbital meningioma. Surgery was indicated and the patient was deemed a candidate for an endoscopic transorbital approach. The surgery was discussed in detail with the patient and consent was obtained.

### 2:42 Patient Positioning.

The patient is positioned supine, with the head on a horseshoe head rest.

### 2:46 Skin Incision.

A superior palpebral incision is made along the patient’s superior palpebral sulcus and extended laterally to expose the zygomatic bone.

### 2:53 Exposure.

A subperiosteal plane is developed and deepened, immediately exposing the hyperostotic bone of the lateral orbital wall. A diamond drill is used to remove the hyperostotic bone, first exposing the temporalis fascia, which serves as our lateral border. Dissectors and forceps are also used intermittently to remove bony fragments. This exposes the middle fossa dura and sagittal crest,^10^ which in this case is hyperostotic and adherent to the periorbita. It is slowly and carefully dissected to avoid fat herniation into the surgical field. The sagittal crest is removed, and drilling is continued superiorly until the lateral anterior fossa dura is encountered.

### 3:33 Optic Nerve Decompression.

Our attention then turns medially to expose the orbital roof and begin optic nerve decompression. Unroofing of the orbit begins with the diamond drill, until the meningo-orbital band needs to be coagulated and cut to facilitate exposure. Further drilling of the orbital roof exposes the medial anterior fossa dura. Neuronavigation is used to confirm that we are at the base of the anterior clinoid. Drilling is continued until the superior lateral optic nerve within the optic canal is identified, along with most important anatomical landmarks, namely the superior orbital fissure, as well as anterior and middle fossae dura. The lesser sphenoid wing is removed, exposing the anterior and middle fossae.

### 4:40 Anterior Clinoidectomy.

Drilling is once again directed medially toward the anterior clinoid, which is emptied and removed. This is achieved by sequentially using the diamond drill within the anterior clinoid, dissectors to separate bony fragments for the dura, and forceps to remove them. Neuronavigation is used to confirm that only the tip of the anterior clinoid was left in place to avoid neurovascular injury and ensure proper optic nerve decompression, which was the objective of the surgery.

### 5:15 Meningioma Resection.

Next, the exposed dura of the middle fossa is extensively coagulated and cut around the meningioma. This allows the dura and meningioma to be lifted off the surface of the brain. Remaining dural attachments are identified, coagulated, and cut, allowing en bloc resection of most of the lesion. A curette is used to remove remaining tumor. The edges of the dura are coagulated when necessary. During this step, the temporal pole underlying the dural opening can clearly be seen and is free from tumor residue.

### 5:55 Reconstruction and Closure.

Reconstruction begins with an inlay of dura patch. Fat is added to the edges of the dural opening, and a fascia lata graft is used as an overlay with Tisseel.

### 6:13 Postoperative Course.

Postoperative imaging revealed subtotal resection of the spheno-orbital meningioma and adequate optic nerve decompression. Notably, there remains a certain degree of proptosis, which decreased from 23 to 18 mm, as can be seen on the postoperative imaging. An alternative option to further improve proptosis reduction is an endoscopic endonasal optic nerve decompression with posterior third medial orbital decompression. Postoperatively, the patient developed transient third to sixth cranial nerve palsies, which fully recovered. The patient had significant improvement of her vision in the left eye, achieving 20/200 vision.

### 6:50 Discussion.

Several advantages of endoscopic transorbital approaches are noteworthy and particularly relevant to this case. This approach offers a direct, shorter distance to target which follows the axis of the middle fossa and avoids neurovascular structures. Less brain retraction and temporal muscle manipulation are necessary, and the procedure leaves only a barely visible scar on the superior eyelid. Lastly, shorter hospitalizations can be achieved, and more comorbid patients can benefit from surgery. However, surgeons must remain mindful of the challenges of this approach, which include a steep learning curve due to new technique and anatomy, a smaller corridor with associated reduced working space, and the possibility of orbital apex syndrome, especially in larger tumors.^6,7^ The latter can possibly be avoided with orbital osteotomies, which reduce retraction of orbit.

### 7:40 Conclusions.

In conclusion, endoscopic transorbital procedures are effective in the treatment of lesions of the middle fossa. They offer a minimally invasive approach with a barely visible scar and minimal brain retraction. These approaches allow access to previously mentioned structures in a more direct trajectory than a pterional or endonasal approach. Multilayered reconstruction and gentle orbit retraction are necessary to avoid complications. Although the learning curve can be steep, as with all minimally invasive surgeries, we advocate that endoscopic transorbital approaches through the eyelid are an alternative to traditional frontotemporal craniotomies in the surgical management of middle fossa pathology. Thank you.

## Disclosures

The authors report no conflict of interest concerning the materials or methods used in this study or the findings specified in this publication.

## Author Contributions

Primary surgeon: Lavergne. Assistant surgeon: Brunette-Clement, Kalin-Hajdu. Editing and drafting the video and abstract: Brunette-Clement. Critically revising the work: Brunette-Clement, Lavergne. Reviewed submitted version of the work: Brunette-Clement, Lavergne. Approved the final version of the work on behalf of all authors: Brunette-Clement. Supervision: Lavergne.

## Supplemental Information

### Previous Presentations

A shorter version of this case was presented as a video presentation at the 33rd Annual Meeting of the North American Skull Base Society, Atlanta, GA, February 16–18, 2024.

### Patient Informed Consent

The necessary patient informed consent was obtained in this study.
